# Supplementary material for: Fossil ribcages of Homo sapiens provide new insights into modern human evolution
Source: Commun Biol. 2025 Jul 10;8:1038. doi: 10.1038/s42003-025-08472-3 (PMC12246208; doi:10.1038/s42003-025-08472-3)
Supplement: Supplementary file 2 — Supplementary Information [file 42003_2025_8472_MOESM2_ESM.pdf]

SUPPLEMENTARY INFORMATION

Title: Fossil ribcages of *Homo sapiens* provide new insights into modern human evolution

Authors: José M. López-Rey[1,2,*], Isabelle Crevecoeur[3], Hila May[4,5], Dani Nadel[6], Carlos A. Palancar[1], Marta Gómez-Recio[1,2], Daniel García-Martínez[7,8,9], & Markus Bastir[1]

Affiliations

[1] Paleoanthropology Group, Department of Paleobiology, Museo Nacional de Ciencias Naturales (MNCN-CSIC), Calle José Gutiérrez Abascal, 2, 28006 Madrid, Spain

[2] Department of Biology, Faculty of Sciences, Universidad Autónoma de Madrid (UAM), Calle Darwin, 2, 28049 Madrid, Spain

[3] UMR 5199 PACEA, CNRS, Université de Bordeaux, Pessac Cedex, France

[4] Department of Anatomy and Anthropology, Gray Faculty of Medical & Health Sciences, Tel Aviv University, Tel Aviv-Yafo, Israel

[5] Shmunis Family Anthropology Institute, the Dan David Center for Human Evolution and Biohistory Research, Gray Faculty of Medical & Health Sciences, Tel Aviv University, Tel Aviv-Yafo, Israel

[6] Zinman Institute of Archaeology, University of Haifa, Mount Carmel, Haifa, Israel

[7] Physical Anthropology Unit, Faculty of Biological Sciences, Universidad Complutense de Madrid (UCM), Calle José Antonio Novais, 12, 28040 Madrid, Spain

[8] Center for Functional Ecology - Science for People and the Planet (CFE). Laboratory of Forensic Anthropology, Centre for Functional Ecology, Department of Life Sciences, University of Coimbra (UC), Calçada Martim de Freitas, 3000-456 Coimbra, Portugal

[9] Centro Nacional de Investigación sobre la Evolución Humana (CENIEH), Paseo de la Sierra de Atapuerca 3, 09002 Burgos, Spain

***Corresponding author:** J. M. López-Rey (jolopezr@mncn.csic.es)

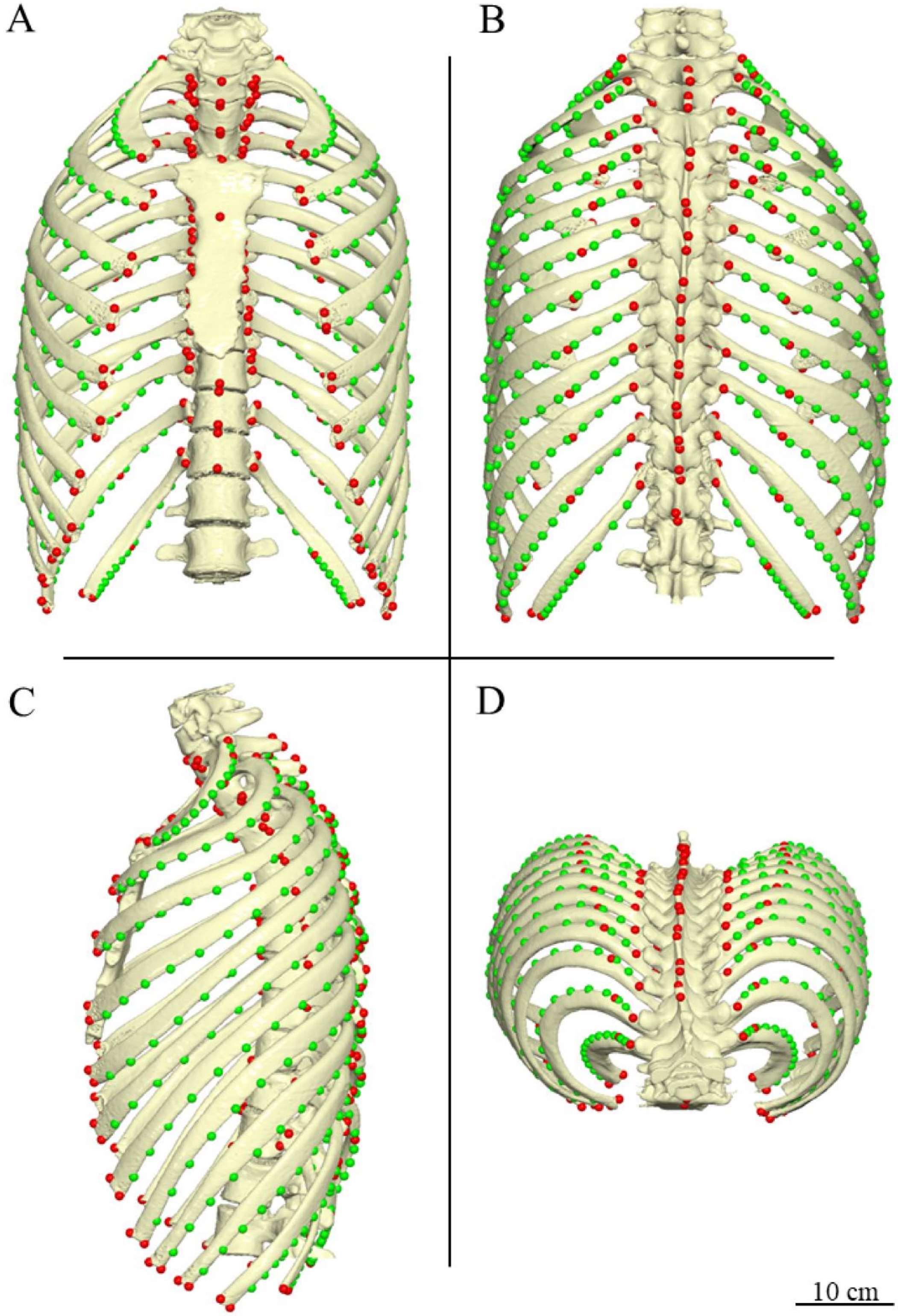

Supplementary Fig. 1. Protocol of landmarks and semilandmarks employed in this study, including both ribs and vertebrae. The template is shown in A) frontal, B) dorsal, C) lateral, and D) cranial view.

Supplementary Table 1. Description of the landmarks and semilandmarks included in the template

Region	Number	Name	Category
Sternum	1	upper sternal notch	Landmark
	2	manubrium to upper sternal corpus	
Rib outline	3 to 18	rib 1 left point 1 to 15	Landmark (1, 15) Semilandmark (2-14)
	19 to 32	rib 1 right point 1 to 15	
	33 to 47	rib 2 left point 1 to 15	
	48 to 62	rib 2 right point 1 to 15	
	63 to 77	rib 3 left point 1 to 15	
	78 to 92	rib 3 right point 1 to 15	
	93 to 107	rib 4 left point 1 to 15	
	108 to 122	rib 4 right point 1 to 15	
	123 to 137	rib 5 left point 1 to 15	
	138 to 152	rib 5 right point 1 to 15	
	153 to 167	rib 6 right point 1 to 15	
	168 to 182	rib 6 left point 1 to 15	
	183 to 197	rib 7 right point 1 to 15	
	198 to 212	rib 7 left point 1 to 15	
	213 to 227	rib 8 right point 1 to 15	
	228 to 242	rib 8 left point 1 to 15	
	243 to 257	rib 9 right point 1 to 15	
	258 to 272	rib 9 left point 1 to 15	
	273 to 287	rib 10 right point 1 to 15	
	288 to 302	rib 10 left point 1 to 15	
	403 to 417	rib 11 left point 1 to 15	
	418 to 432	rib 11 right point 1 to 15	
	433 to 447	rib 12 left point 1 to 15	
	448 to 462	rib 12 right point 1 to 15	
Ribhead	303	rib 1 right superior ribhead	Landmark
	304	rib 1 right inferior rib head	
	305	rib 1 right ant interart crest ribhead	
	306	rib 1 right inf costal angle	
	307	rib 1 right sup point rib end	
	308	rib 1 left superior ribhead	
	309	rib 1 left inferior ribhead	
	310	rib 1 left ant interart crest ribhead	
	311	rib 1 left inf costal angle	
	312	rib 1 left sup point rib end	
	313	rib 2 right superior ribhead	
	314	rib 2 right inferior rib head	

	315	rib 2 right ant interart crest ribhead	
	316	rib 2 right inf costal angle	
	317	rib 2 right sup point rib end	
	318	rib 2 left superior ribhead	
	319	rib 2 left inferior ribhead	
	320	rib 2 left ant interart crest ribhead	
	321	rib 2 left inf costal angle	
	322	rib 2 left sup point rib end	
	323	rib 3 right superior ribhead	
	324	rib 3 right inferior rib head	
	325	rib 3 right ant interart crest ribhead	
	326	rib 3 right inf costal angle	
	327	rib 3 right sup point rib end	
	328	rib 3 left superior ribhead	
	329	rib 3 left inferior ribhead	
	330	rib 3 left ant interart crest ribhead	
	331	rib 3 left inf costal angle	
	332	rib 3 left sup point rib end	
	333	rib 4 right superior ribhead	
	334	rib 4 right inferior rib head	Landmark
	335	rib 4 right ant interart crest ribhead	
	336	rib 4 right inf costal angle	
	337	rib 4 right sup point rib end	
	338	rib 4 left superior ribhead	
	339	rib 4 left inferior ribhead	
	340	rib 4 left ant interart crest ribhead	
	341	rib 4 leftinf costal angle	
	342	rib 4 left sup point rib end	
	343	rib 5 right superior ribhead	
	344	rib 5 right inferior rib head	
	345	rib 5 right ant interart crest ribhead	
	346	rib 5 right inf costal angle	
	347	rib 5 right sup point rib end	
	348	rib 5 left superior ribhead	
	349	rib 5 left inferior ribhead	
	350	rib 5 left ant interart crest ribhead	
	351	rib 5 left inf costal angle	
	352	rib 5 left sup point rib end	
	353	rib 6 right superior ribhead	
	354	rib 6 right inferior rib head	
	355	rib 6 right ant interart crest ribhead	

	356	rib 6 right inf costal angle	
	357	rib 6 right sup point rib end	
	358	rib 6 left superior ribhead	
	359	rib 6 left inferior ribhead	
	360	rib 6 left ant interart crest ribhead	
	361	rib 6 left inf costal angle	
	362	rib 6 left sup point rib end	
	363	rib 7 right superior ribhead	
	364	rib 7 right inferior rib head	
	365	rib 7 right ant interart crest ribhead	
	366	rib 7 right inf costal angle	
	367	rib 7 right sup point rib end	
	368	rib 7 left superior ribhead	
	369	rib 7 left inferior ribhead	
	370	rib 7 left ant interart crest ribhead	
	371	rib 7 left inf costal angle	
	372	rib 7 left sup point rib end	
	373	rib 8 right superior ribhead	
	374	rib 8 right inferior rib head	
	375	rib 8 right ant interart crest ribhead	Landmark
	376	rib 8 right inf costal angle	
	377	rib 8 right sup point rib end	
	378	rib 8 left superior ribhead	
	379	rib 8 left inferior ribhead	
	380	rib 8 left ant interart crest ribhead	
	381	rib 8 left inf costal angle	
	382	rib 8 left sup point rib end	
	383	rib 9 right superior ribhead	
	384	rib 9 right inferior rib head	
	385	rib 9 right ant interart crest ribhead	
	386	rib 9 right inf costal angle	
	387	rib 9 right sup point rib end	
	388	rib 9 left superior ribhead	
	389	rib 9 left inferior ribhead	
	390	rib 9 left ant interart crest ribhead	
	391	rib 9 left inf costal angle	
	392	rib 9 left sup point rib end	
	393	rib 10 right superior ribhead	
	394	rib 10 right inferior rib head	
	395	rib 10 right ant interart crest ribhead	
	396	rib 10 right inf costal angle	

	397	rib 10 right sup point rib end	
	398	rib 10 left superior ribhead	
	399	rib 10 left inferior ribhead	
	400	rib 10 left ant interart crest ribhead	
	401	rib 10 left inf costal angle	
	402	rib 10 left sup point rib end	
	503	rib 11 left superior ribhead	
	504	rib 11 left inferior ribhead	
	505	rib 11 left inf costal angle	
	506	rib 11 left sup point rib end	
	507	rib 11 right superior ribhead	
	508	rib 11 right inferior ribhead	Landmark
	509	rib 11 right inf costal angle	
	510	rib 11 right sup point rib end	
	511	rib 12 left superior ribhead	
	512	rib 12 left inferior ribhead	
	513	rib 12 left inf costal angle	
	514	rib 12 left sup point rib end	
	515	rib 12 right superior ribhead	
	516	rib 12 right inferior ribhead	
	517	rib 12 right inf costal angle	
	518	rib 12 right sup point rib end	
	463	T1 upper anterior	
	464	T1 lower anterior	
	465	T2 upper anterior	
	466	T2 lower anterior	
	467	T3 upper anterior	
	468	T3 lower anterior	
	469	T4 upper anterior	
	470	T4 lower anterior	
	471	T5 upper anterior	
Vertebrae	472	T5 lower anterior	Landmark
	473	T6 upper anterior	
	474	T6 lower anterior	
	475	T7 upper anterior	
	476	T7 lower anterior	
	477	T8 upper anterior	
	478	T8 lower anterior	
	479	T9 upper anterior	
	480	T9 lower anterior	
	481	T10 upper anterior	

482	T10 lower anterior	
483	T1 upper posterior	
484	T1 lower posterior	
485	T2 upper posterior	
486	T2 lower posterior	
487	T3 upper posterior	
488	T3 lower posterior	
489	T4 upper posterior	
490	T4 lower posterior	
491	T5 upper posterior	
492	T5 lower posterior	
493	T6 upper posterior	
494	T6 lower posterior	
495	T7 upper posterior	Landmark
496	T7 lower posterior	
497	T8 upper posterior	
498	T8 lower posterior	
499	T9 upper posterior	
500	T9 lower posterior	
501	T10 upper posterior	
502	T10 lower posterior	
519	T11 upper posterior	
520	T11 lower posterior	
521	T12 upper posterior	
522	T12 lower posterior	
523	T11 upper anterior	
524	T11 lower anterior	
525	T12 upper anterior	
526	T12 lower anterior	
