# Supplementary material for: Fossil ribcages of Homo sapiens provide new insights into modern human evolution
Source: Commun Biol. 2025 Jul 10;8:1038. doi: 10.1038/s42003-025-08472-3 (PMC12246208; doi:10.1038/s42003-025-08472-3)
Supplement: Supplementary file 3 — Description of Additional Supplementary Files [file 42003_2025_8472_MOESM3_ESM.pdf]

Description of Additional Supplementary Files

File name: Supplementary Data 1-2

Description: Numerical source data for plotting the UPGMA cluster and the PCA
