# Supplementary material for: Fossil ribcages of Homo sapiens provide new insights into modern human evolution
Source: Commun Biol. 2025 Jul 10;8:1038. doi: 10.1038/s42003-025-08472-3 (PMC12246208; doi:10.1038/s42003-025-08472-3)
Supplement: Supplementary file 4 — Supplementary Data 1 [file 42003_2025_8472_MOESM4_ESM.pdf]

Supplementary Data 1. Distance matrix between groups based on differences in centroid size, calculated through permutation tests (10,000). This matrix was used as input for the hierarchical UPGMA cluster shown in **Fig. 2**.

Groups	*H. sapiens* - Cold	Dolní Věstonice 13	KNM-WT 15000	Nazlet Khater 2	Neanderthals	Ohalo II H2	Ötzi	*H. sapiens* - Temperate
Dolní Věstonice 13	138.18	-	-	-	-	-	-	-
KNM-WT 15000	190.96	329.14	-	-	-	-	-	-
Nazlet Khater 2	76.48	214.66	114.49	-	-	-	-	-
Neanderthals	242.27	104.09	433.23	318.85	-	-	-	-
Ohalo II H2	54.02	192.21	136.93	22.45	296.29	-	-	-
Ötzi	40.52	97.66	231.48	116.99	201.75	94.54	-	-
H. sapiens - Temperate	42.64	180.82	148.32	33.84	284.91	11.38	83.16	-
H. sapiens - Warm	182.69	320.88	8.26	106.22	424.98	128.67	223.22	140.06
