# Supplementary material for: Fossil ribcages of Homo sapiens provide new insights into modern human evolution
Source: Commun Biol. 2025 Jul 10;8:1038. doi: 10.1038/s42003-025-08472-3 (PMC12246208; doi:10.1038/s42003-025-08472-3)
Supplement: Supplementary file 5 — Supplementary Data 2 [file 42003_2025_8472_MOESM5_ESM.pdf]

Supplementary Data 2. Principal component (PC)1 and PC2 scores extracted from the PCA in shape space performed on the sample. These values were plotted and visualized in **Fig. 3**.

ID	PC1 scores	PC2 scores
KNM-WT 15000	-0.082419008	0.044165329
Kebara 2	-0.033722605	0.026285071
Shanidar 3	0.016085216	-0.002684898
Nazlet Khater 2	0.000716395	0.000678329
Dolní Věstonice 13	-0.069844913	0.025601818
Ohalo II	-0.002617754	-0.00117337
Ötzi	-0.016120703	-0.008514543
KAL-0924	0.047448249	0.049739453
KAL-0926	0.006438459	0.06749601
AMNH 99.1_400	0.008646043	0.018476839
AMNH 99.1_481	0.035473692	0.018482081
AMNH 99.1_542	0.087232509	-0.066097192
KAL-0151	0.066539837	0.010256111
KAL-0152	0.043574615	0.02200382
KAL-0041	-0.022613873	-0.032745259
418	-0.128921719	-0.074869059
ED 10	-0.014471638	-0.028117956
417	-0.044095458	0.018230759
Las Mandíbulas	-0.016614969	0.026099639
ACC 27	-0.034703345	-0.033312574
ACC 77	0.012458215	-0.071670738
ACC 58	0.006293074	-0.017207529
B_1030_34982	0.024454789	-0.069997112
B_1031_34983	0.059284956	-0.040928836
B_1062_35014	-0.013682542	-0.007879417
119259	-0.074082394	-0.036671712
170124	-0.067206105	0.026362456
184880	-0.080507279	-0.025864736
106127	-0.064365647	0.009730622
124031	-0.060635281	-0.036725041
161103	-0.069534907	-0.061284944
B_322_25472	-0.031636327	0.066723543
B_353_25478	-0.001600532	-0.003365229
B_278_25463	-0.003118415	0.009379173
B_249_12262	0.08052273	-0.030755926
B_252_25503	-0.017207365	0.033132546
B_316_12260	0.07523182	0.009804101
B_348_12267	0.017865303	-0.010132429
B_368_20820-1	0.033109999	-0.052369531
B_378_24990	0.027454394	-0.018931147

B_395bis_24985	0.008809335	0.024649222
B_133_11247	0.017831263	-0.008690543
B_136_12245-2	-0.012925299	0.023572333
B_136_12246-2	0.049660834	0.002829639
B_273_11248	0.036845049	-0.020770699
B_273_11249	0.054340606	-0.018066965
B_15_23876	0.023526682	-0.028259238
B_318_23873	0.031434586	0.018326462
112	0.014610062	0.037760569
166	0.037223518	0.004644366
192	0.065212938	0.008168576
25	0.088519013	-0.033167367
77	0.070733063	-0.035576795
107	-0.013867451	-0.018822098
100139	-0.069696534	-0.009778124
103805	-0.038358131	-0.039528181
111819	-0.060340022	-0.113552124
B_86_9778	0.047207829	0.036815282
B_87_9779	-0.004810548	0.107697515
B_88_9776	0.006340372	-0.014339291
B_147_8274	-0.030034722	0.087554907
B_150_8278	0.023569665	0.029072873
B_169_8277	-0.007501654	0.040729509
B_117_23943	0.004535594	0.028159513
B_121_19855	-0.025345989	0.095778117
B_328bis_19852	-0.016627574	0.043444022
