# Supplementary material for: Fossil ribcages of Homo sapiens provide new insights into modern human evolution
Source: Commun Biol. 2025 Jul 10;8:1038. doi: 10.1038/s42003-025-08472-3 (PMC12246208; doi:10.1038/s42003-025-08472-3)
Supplement: Supplementary file 6 — Reporting summary [file 42003_2025_8472_MOESM6_ESM.pdf]

nature portfolio

Corresponding author(s): José M. López-Rey

Last updated by author(s): 17/06/2025

Reporting Summary

Nature Portfolio wishes to improve the reproducibility of the work that we publish. This form provides structure for consistency and transparency in reporting. For further information on Nature Portfolio policies, see our Editorial Policies and the Editorial Policy Checklist.

Statistics

For all statistical analyses, confirm that the following items are present in the figure legend, table legend, main text, or Methods section.

n/a	Confirmed	
☐	☒	The exact sample size (*n*) for each experimental group/condition, given as a discrete number and unit of measurement
☒	☐	A statement on whether measurements were taken from distinct samples or whether the same sample was measured repeatedly
☐	☒	The statistical test(s) used AND whether they are one- or two-sided<br>*Only common tests should be described solely by name; describe more complex techniques in the Methods section.*
☐	☒	A description of all covariates tested
☐	☒	A description of any assumptions or corrections, such as tests of normality and adjustment for multiple comparisons
☐	☒	A full description of the statistical parameters including central tendency (e.g. means) or other basic estimates (e.g. regression coefficient) AND variation (e.g. standard deviation) or associated estimates of uncertainty (e.g. confidence intervals)
☐	☒	For null hypothesis testing, the test statistic (e.g. *F*, *t*, *r*) with confidence intervals, effect sizes, degrees of freedom and *P* value noted<br>*Give P values as exact values whenever suitable.*
☒	☐	For Bayesian analysis, information on the choice of priors and Markov chain Monte Carlo settings
☐	☒	For hierarchical and complex designs, identification of the appropriate level for tests and full reporting of outcomes
☒	☐	Estimates of effect sizes (e.g. Cohen's *d*, Pearson's *r*), indicating how they were calculated

Our web collection on statistics for biologists contains articles on many of the points above.

Software and code

Policy information about availability of computer code

Data collection	Data collection was done in Artec Studio 16 (processing 3D models), LhpFusionBox (3D reconstructions), and Viewbox 4.1 (geometric morphometrics)
Data analysis	Data analysis was done in RStudio v. 2023.12.1-402

For manuscripts utilizing custom algorithms or software that are central to the research but not yet described in published literature, software must be made available to editors and reviewers. We strongly encourage code deposition in a community repository (e.g. GitHub). See the Nature Portfolio guidelines for submitting code & software for further information.

Data

Policy information about availability of data

All manuscripts must include a data availability statement. This statement should provide the following information, where applicable:

- Accession codes, unique identifiers, or web links for publicly available datasets
- A description of any restrictions on data availability
- For clinical datasets or third party data, please ensure that the statement adheres to our policy

Costovertebral image data must be requested from the corresponding housing institutions and curators. The R script including all analyses and figure preparations is available from the corresponding author upon request. Other kinds of data that support the findings of this study are available from the corresponding author to any researcher for purposes of reproducing or extending the analyses.

Research involving human participants, their data, or biological material

Policy information about studies with human participants or human data. See also policy information about sex, gender (identity/presentation), and sexual orientation and race, ethnicity and racism.

Reporting on sex and gender	The four fossils studied in this research were identified as male. Therefore, we selected a control sample of male individuals to ensure consistency. Gender identity was not relevant.
Reporting on race, ethnicity, or other socially relevant groupings	The study includes individuals from a worldwide distribution. Therefore, we specify the population they belong to and their geographic origin.
Population characteristics	Not applicable.
Recruitment	Most of the sample came from surface scans of individuals from osteo-archaeological collections, while a few were CT scans from the New Mexico Decedent Image Database (NMDID).
Ethics oversight	We affirm that samples were collected in a responsible manner and in accordance with relevant permits and local laws.

Note that full information on the approval of the study protocol must also be provided in the manuscript.

Field-specific reporting

Please select the one below that is the best fit for your research. If you are not sure, read the appropriate sections before making your selection.

☐ Life sciences ☐ Behavioural & social sciences ☒ Ecological, evolutionary & environmental sciences

For a reference copy of the document with all sections, see nature.com/documents/nr-reporting-summary-flat.pdf

Ecological, evolutionary & environmental sciences study design

All studies must disclose on these points even when the disclosure is negative.

Study description	The aim of this research is collecting a costovertebral sample of fossil H. sapiens from the Upper Pleistocene onwards to elucidate not only whether their reconstructed ribcages fall within the recent human range of variation, but also the potential relationship between their morphology and the location and ancestry of each specimen.
Research sample	Individuals studied in this article can be divided into three categories: fossil H. sapiens, recent H. sapiens and other fossil Homo. The first group includes costovertebral material from Nazlet Khater 2, Dolní Věstonice 13, Ohalo II H2, and Ötzi. Comparative recent H. sapiens sample consisted of 59 individuals from 19 populations distributed along the five inhabited continents. Eventually, we studied the ribcages of three fossil Homo: KNM-WT 15000 (H. erectus s.l.) Kebara 2 and Shanidar 3 (Neanderthals).
Sampling strategy	Given the difficulty of accessing human remains, particularly costovertebral material, our sampling strategy was to gather as many individuals as possible.
Data collection	Data collection for the control sample was primarily performed using surface scanners by the authors of the research. Only a few individuals were requested from the NMDID due to the difficulty of accessing osteo-archaeological material from certain populations. Scans of fossils were requested from the corresponding host institutions.
Timing and spatial scale	Sampling was conducted in two phases. First, some individuals were surface-scanned in 2018. Then, the majority of the individuals studied were either scanned or kindly requested in 2023.
Data exclusions	Not applicable.
Reproducibility	Once ribcages were 3D reconstructed, measurements were taken using a template of landmarks and semilandmarks that can be easily reproduced.
Randomization	Fossil individuals, except for Neanderthals, were studied separately. The control sample was grouped according to the latitude of population origin.
Blinding	All individuals included in the study were anonymous.

Did the study involve field work? ☐ Yes ☒ No

Reporting for specific materials, systems and methods

We require information from authors about some types of materials, experimental systems and methods used in many studies. Here, indicate whether each material, system or method listed is relevant to your study. If you are not sure if a list item applies to your research, read the appropriate section before selecting a response.

Materials & experimental systems

n/a	Involved in the study
☒	☐ Antibodies
☒	☐ Eukaryotic cell lines
☐	☒ Palaeontology and archaeology
☒	☐ Animals and other organisms
☒	☐ Clinical data
☒	☐ Dual use research of concern
☒	☐ Plants

Methods

n/a	Involved in the study
☒	☐ ChIP-seq
☒	☐ Flow cytometry
☒	☐ MRI-based neuroimaging

Palaeontology and Archaeology

Specimen provenance

Individuals include in this study come from the paleoanthropological collections of 11 different institutions. The list of institutions is: American Museum of Natural History (N.Y.C., U.S.A.), University of Copenhagen (Denmark), Universidad del Centro de la Provincia de Buenos Aires (Argentina), Museo Nacional de Historia Natural de Chile (Santiago, Chile), Musée de l'Homme (Paris, France), Universidad Complutense de Madrid (Spain), DRYAS Octopetala (Coimbra, Portugal), Kenya National Museum (Nairobi, Kenya), Tel Aviv University (Israel), Smithsonian Institution (Washington D.C., U.S.A.), New Mexico Decedent Image Database (NMDID).

Specimen deposition

Specimens must be requested to the host institutions.

Dating methods

Dating of specimens was provided by the host institutions.

☐ Tick this box to confirm that the raw and calibrated dates are available in the paper or in Supplementary Information.

Ethics oversight

We affirm that samples were collected in a responsible manner and in accordance with relevant permits and local laws.

Note that full information on the approval of the study protocol must also be provided in the manuscript.

Plants

Seed stocks

Not applicable

Novel plant genotypes

Not applicable

Authentication

Not applicable
